# Human Connections and Their Roles in the Occupational Well-being of Healthcare Professionals: A Study on Loneliness and Empathy

**DOI:** 10.3389/fpsyg.2017.01475

**Published:** 2017-08-29

**Authors:** Jorge Soler-Gonzalez, Montserrat San-Martín, Roberto Delgado-Bolton, Luis Vivanco

**Affiliations:** ^1^Faculty of Medicine (GESEC Group), University of Lleida Lleida, Spain; ^2^Institut de Recerca Biomèdica de Lleida (IRB), IDIAP Jordi Gol, Institut Català de la Salut (ICS) Lleida, Spain; ^3^Faculty of Social Sciences of Melilla, University of Granada Melilla, Spain; ^4^Scientific Computing Group (GRUCACI), University of La Rioja Logroño, Spain; ^5^Platform of Bioethics and Medical Education, Center for Biomedical Research of La Rioja Logroño, Spain; ^6^Education Committee Board, University Hospital San Pedro Logroño, Spain; ^7^Department of Nuclear Medicine, Center for Biomedical Research of La Rioja Logroño, Spain; ^8^National Centre of Documentation on Bioethics Logroño, Spain

**Keywords:** empathy, loneliness, somatization, exhaustion, alienation, healthcare professionals

## Abstract

Human connections are key to the promotion of health and prevention of illness; moreover, illness can cause deterioration of human connections. Healthcare professional–patient relationships are key to ensuring the preservation of adequate human connections. It is important for healthcare professionals to develop their ability to foster satisfactory human connections because: (i) they represent social support for patients; and (ii) they prevent work-related stress. In this study we assessed the relationship between absence (loneliness) and presence (empathy) of human connections with the occupational well-being of healthcare professionals. The Scale of Collateral Effects, which measures somatization, exhaustion, and work alienation; the Jefferson Scale of Empathy; and the Social and Emotional Loneliness Scale for Adults, were mailed to 628 healthcare professionals working in Spanish public healthcare institutions. The following explanatory variables were used to evaluate work well-being: (a) empathy, as a professional competence; (b) loneliness, age, and family burden, as psychological indicators; and (c) professional experience, work dedication, and salary, as work indicators. Comparison, correlation, and regression analyses were performed to measure the relationships among these variables and occupational well-being. Of 628 surveys mailed, 433 (69% response rate) were returned fully completed. Adequate reliability was confirmed for all instruments. The entire sample was divided into four groups, based on the combined variable, “occupation by sex.” Comparative analyses demonstrated differences among “occupation by sex” groups in collateral effects (*p* = 0.03) and empathy (*p* = 0.04), but not loneliness (*p* = 0.84). Inverse associations between empathy and collateral effects were confirmed for somatization (*r* = -0.16; *p* < 0.001), exhaustion (*r* = -0.14; *p* = 0.003), and work alienation (*r* = -0.16; *p* < 0.001). Furthermore, loneliness was positively associated with collateral effects (*r* = 0.22; *p* < 0.001). Neither family burden, nor work dedication to clinics or management activities were associated with the three collateral effects measured. These findings support an important role for empathy in the prevention of work stress in healthcare professionals. They also confirm that loneliness, as a multidimensional and domain specific experience, is detrimental to occupational well-being.

## Introduction

In his book *“Social: Why our brains are wired to connect,”* Lieberman (2013) compiled extensive research evidence demonstrating the importance of human connections for human beings as a core element of human nature. According to Lieberman, human connections are an essential part of the welfare of our societies, due to their roles in the promotion of health and the prevention of physical and mental illness. However, this fragile and poorly understood aspect of our lives can be influenced by individual and environmental factors capable of deteriorating the capacity of individuals to establish and to maintain human connections. Illness is certainly one such factor.

In patients with chronic disease, the progressive lack of positive human connections increases their vulnerability to suffering from loneliness and social isolation, with negative effects on their health conditions (Steptoe et al., 2013; Neufeld et al., 2015; Mann et al., 2017). Therefore, it is reasonable to assume that, in such patients, healthcare professional–patient relationships tend to fill this social need. In addition, for healthcare professionals, the ability to forge satisfactory human connections is also important, not only because of the role they play as social support for their patients (Decety and Fotopoulou, 2014; Hojat, 2016), but also because of their protective role against work-related stress (Melamed et al., 2001; Rogers et al., 2016; Marilaf-Caro et al., 2017); the latter aspect (i.e., the role that positive human connections play in the workplace) provides a fascinating and less explored field of study.

According to Dutton and Ragins (2007), too often work and work relationships are not included in lists of things that make life worth living. Paradoxically, people spend much of their time at work and, consequently, work relationships become central, not only for how work gets done, but also for the quality of their lives. In the words of these researchers, those relationships “can be a generative source of enrichment, vitality, and learning that helps individuals, groups, and organizations grow, thrive, and flourish” (Dutton and Ragins, 2007, p. 3). Such relationships become even more meaningful when they develop in stressful situations. This has been demonstrated in organizations that are exposed to stressful situations daily, such as hospitals (Gittell, 2008; Gittell et al., 2013). However, this phenomenon has also been reported in organizations exposed to unexpected adverse events (Powley, 2009). In both cases, the ability to develop (or not) positive human connections has been identified as an important factor influencing the well-being of workers and their professional performance.

Defined as the perception that one lacks meaningful connections with others, loneliness is a complex phenomenon that is an outcome of numerous factors related to unsatisfactory human connections at different levels (Weiss, 1973, 1987; DiTommaso and Spinner, 1997). According to different authors, several factors influence loneliness including: early child rearing environments or insecure attachment relationships in childhood (Hojat, 1987; Shaver and Hazan, 1987); dysfunctional social networks and social forces (Jones and Moore, 1987); non-facilitative living environments (DiTommaso and Spinner, 1997); or even a lack of interpersonal skills (Hojat, 2016). It is not a coincidence that many of these factors also contribute to a deficient capacity for empathy, as has been demonstrated by some studies of students of medicine (Hojat et al., 2005) and nursing (Cunico et al., 2012; Domínguez et al., 2017), and more recently healthcare professionals (Marilaf-Caro et al., 2017).

In the general population, loneliness is closely associated with depression and similar mood state disruptions (Young, 1982; Hojat, 1983; Gerstein et al., 1987; Heinrich and Gullone, 2006). It has also been associated with behavioral and mental health problems, including depression, anxiety, neuroticism, tough-mindedness, low self-esteem, and even suicidal ideation (Hojat, 1983; DiTommaso and Spinner, 1997; Mellor et al., 2008). According to DiTommaso and Spinner (1997), loneliness is also associated with a number of psychosomatic symptoms, including headaches, poor appetite, fatigue, and poor physical health. Loneliness not only acts as a barrier against psychological well-being, but also has a negative effect on physical health through its effects on the immune system (Kennedy et al., 1988). Finally, people who have experienced loneliness are likely to score low on measures of positive aspects of personality conducive to relationship building (Mellor et al., 2008; Salimi, 2011). Disconnected people are less likely to trust others, suggesting that the experience of loneliness is not conducive to forming empathetic relationships (Hojat, 1982). Consistent with this finding, one study reported that impaired peer relationships during medical school could predict later disciplinary action by medical boards against physicians (Papadakis et al., 2005). Thus, the capacity to connect can have a lasting effect on the professional behavior of physicians.

In contrast to loneliness, the establishment of satisfactory (also referred to as positive) human connections has healing power. The association between social connections and health outcomes has been relatively well demonstrated in different contexts (Bennett et al., 2001; Post, 2005; Prilleltensky and Prilleltensky, 2007). According to Hojat (2016, p. 23), social connections are protective and prevent illness because the satisfaction that results from human relationships is an important health-promoting factor. In support of this, some authors (Greenberg et al., 2001) suggest that to have satisfactory human connections (as a consequence of developing empathetic relationships) helps to strengthen self-esteem and liberates an individual from social isolation and experiencing loneliness. This issue is especially important in clinical contexts, where patients are more vulnerable to suffering from isolation and loneliness. Taking this into consideration, for patients to perceive an empathetic relationship with their healthcare providers is an invaluable support during treatment. In contrast, for healthcare professionals, empathy has been described as a central attribute of humanistic medicine (Arnold, 2002), and one of the core elements of professionalism in patient care (Veloski and Hojat, 2006; Vivanco and Delgado-Bolton, 2015). According to Hojat (2016, p. 74), for healthcare professionals, being empathetic in clinical encounters is defined as a “predominantly *cognitive* (rather than an affective or emotional) attribute that involves an *understanding* (rather than feeling) of experiences, concerns and perspectives of the patient, combined with a capacity to *communicate* this understanding, and an *intention to help.*” In such encounters, developing an empathetic engagement based on the balanced development of the three above-mentioned elements (i.e., understanding abilities, communication skills, and intention to help) also protects from the negative effects caused by an intensive emotional involvement (Hojat, 2016; Marilaf-Caro et al., 2017; Yuguero-Torres et al., 2017). According to some authors (Yuguero-Torres et al., 2015; Hojat, 2016; Marilaf-Caro et al., 2017; San-Martín et al., 2017), empathetic relationships with patients are a type of meaningful interpersonal connection for healthcare professionals, and these connections can serve as a buffer against work dissatisfaction, professional burnout, and work-related stress. Furthermore, in nursing, as a healthcare profession that is oriented to patients’ care, empathy has a central role (Mortier et al., 2016). Therefore, empathy is expected to be a characteristic found in all nurses, independently of their specific role. According to some authors (San-Martín et al., 2017), this would explain why nurses have lesser distraction than the physicians concerning empathy, and why this ability is protective in prevention of work distress. On the other hand, in physicians a higher distraction concerning empathy may be caused by the nature of their clinical and medical roles and the professional duties associated to them. It could explain, for example, why physicians who are working in primary care, pediatrics or psychiatric areas tend to be more empathetic than their colleagues who are working in technical and surgical specialties (Hojat, 2016).

Taking the importance of empathy to the well-being of healthcare workers into consideration, this study was designed with the main purpose of assessing the relationship between absence of satisfactory human connections (measured as loneliness) and the presence of satisfactory human connections (measured as empathy) in the promotion of the occupational well-being of physicians and nurses who share workplaces in Spanish healthcare institutions. Three research hypotheses were tested, the first two to determine the association between positive human connections and occupational well-being, and the third to characterize potential factors that influence the development of empathy and the perception of loneliness and work stress. These research hypotheses were as follows: (i) in healthcare professionals who are in contact with patients, the ability to establish positive human connections with their patients (measured as empathy) protects them from the risk of suffering work-related stress (measured as somatization, exhaustion, and work alienation); (ii) in such professionals, the absence of positive human connections (measured as loneliness) increases the risk of suffering work stress at their workplace (measured as somatization, exhaustion, and work alienation); and (iii) in work teams composed of physicians and nurses, work stress (measured as somatization, exhaustion, and work alienation) and empathetic orientation to patients, but not loneliness, vary according to sex and occupation.

## Materials and Methods

### Participants

The study included a sample of 628 physicians and nurses dedicated to direct patient care who were working in the region of Lleida for the Catalonian Healthcare Institute, a Spanish public healthcare institution that offers healthcare assistance in this area of Catalonia. Lleida is the biggest region of Catalonia, with 1,747 physicians and 2,340 nurses in charge of the healthcare of its 370,000 inhabitants. All participants were invited to take part in the study voluntarily and anonymously.

### Principal Measures

#### Psychometric Instruments

To measure work-related stress, the Scale of Collateral Effects (SCE) from the Questionnaire of General Labor Well-being, was used. The SCE is a psychometrically sound instrument composed of three scales: a 5-item scale that measures “somatization,” a 4-item scale that measures “exhaustion,” and a 4-item scale that measures “work alienation” (Blanch et al., 2010). All scales are composed by items that have to be answered on a 7-point Likert-type scale (1 = never, 7 = always). Possible scores for the SCE range from 13 to 91, with higher scores indicating greater self-perceived effects. Originally, the SCE was applied to university teachers and healthcare professionals (physicians and nurses) who were working in Spanish and Latin American institutions. It was designed with the intention to explore the perception of the above three mentioned elements. The SCE has demonstrated a high reliability and validity. Also, a high positive correlation between the SCE and the Maslach Burnout Inventory has been reported for the scales of “emotional exhaustion” and “depersonalization” (Blanch et al., 2010).

To measure empathetic orientation in clinical encounters, the Jefferson Scale of Empathy (JSE; HP-Version) was used. The JSE includes 20 items that measure the empathetic behavior of physicians and other healthcare professionals in the context of patient care (Hojat et al., 2002; Alcorta-Garza et al., 2016). The JSE is answered on a 7-point Likert scale (1 = strongly disagree, 7 = strongly agree), with higher scores associated with more empathetic behavior in the context of patient care. The JSE has enjoyed broad international attention and it has been described as possibly the most researched and widely used instrument for measuring empathy in clinical settings with more than 42 translated versions used in different territories and cultural contexts (Hojat, 2016).

To measure loneliness perception, the short version of the Social and Emotional Loneliness Scale for Adults (SELSA-S), was used. The SELSA-S consists of 15 items, and produces a total loneliness score, as well as scores for three dimensions of loneliness: “family,” “romantic,” and “social” (DiTommaso et al., 2004). The SELSA-S measures the global self-perception of each dimension of loneliness is measured using a 5-item scale. The SELSA-S is answered in a 7-point Likert scale (1 = strongly disagree, 7 = strongly agree) and higher scores indicate a higher perception of loneliness. The following are sample items from each of the SELSA-S dimensions: “I do not feel satisfied with the friends that I have,” “I feel part of a group of friends” (Social dimension), “I have a romantic partner to whose happiness I contribute,” “I have someone who fulfills my emotional needs” (Romantic dimension), and “I feel alone when I’m with my family,” “I feel close to my family” (Family dimension). The SELSA-S has demonstrated good psychometric properties: concurrent and discriminant validity (DiTommaso et al., 2004). In studies with healthcare professionals and students of nursing, the SELSA-S has shown a high reliability with coefficients closer to 0.90 (Domínguez et al., 2017; Marilaf-Caro et al., 2017).

#### Demographics

Additional information about age, sex, occupation (medicine or nursing), professional experience, salary, family burden, and time devoted to patient care and management activities, was collected through a complementary survey.

### Procedures

From March to April 2015, a web-based (Survey Monkey^®^) anonymous questionnaire, based on the above-mentioned instruments, and a complementary information form were distributed as an email link to healthcare professionals working in all healthcare institutions supported by the administration of the Catalonian Healthcare Institute in the province of Lleida, Spain. The email included a cover letter and a web-link to the survey. Prior to beginning the survey, an information page describing the design and purpose of the study was available, following a general protocol previously approved by an independent ethics committee (Ref. CEICLAR PI 199). At the bottom of this page participants were asked to sign to indicate their informed consent. Only participants who agreed to sign the informed consent were allowed to continue with the survey. A reminder was sent following the same protocol 15 days after the survey had initially been distributed. All responses were automatically collected in a database supported by Survey Monkey^®^. The study was performed in collaboration with the Delegations in Lleida of the Professional Association of Physicians (COMLL) and the Professional Association of Nurses (COILL). The work was carried out in accordance with the Declaration of Helsinki of Ethical Principles for medical research involving human subjects adopted by the World Medical Association. There was no potential risk for participants, and anonymity was guaranteed throughout the process.

### Statistical Assessment

Prior to performing any statistical assessment, internal consistency and reliability were calculated for all psychometric instruments administered using Cronbach’s alpha coefficient. Following the guidelines suggested by the American Educational Research Association, coefficient values higher than 0.7 were considered satisfactory.

For the first two research hypotheses, associations of work stress indicators (somatization, exhaustion, and alienation) and measures of empathy and loneliness, were tested using correlation analysis. To test the third research hypothesis, sex (male and female) and occupation (physician and nurse) were used as explanatory variables, while “collateral effects,” “empathy,” and “loneliness” scores were considered dependent variables. To examine the differences between groups due to the main effects of sex and occupation, subgroups were defined using the combination “occupation by sex.” As the variance differed among the subgroups, and also because the subgroup sizes were unbalanced, a Kruskal–Wallis test was performed. *Post hoc* analyses were performed using the Bonferroni test. Moreover, after analysis of normality, correlation analyses were performed to determine whether elements, other than occupation and sex, influenced the development of empathy and the perception of loneliness and work stress. The other elements analyzed were: age, family burden, professional experience, work dedication, and salary. As professional experience and age can be related variables, when an association with age was confirmed, a partial correlation coefficient was determined for the element assessed. This analysis was performed to measure the degree of association between the correlated element and professional experience, with the effect of the variable age removed. Finally, regression analysis was performed with interaction terms for each of the three collateral effects measured to examine the differences among the “occupation by sex” groups. All analyses were performed using R statistical software, version 3.1.1 for Windows using *multilevel* (Bliese, 2013), *nortest* (Gross, 2012), and *ppcor* (Kim, 2015) packages.

## Results

Of the 628 healthcare professionals who agreed to participate in the study, 433 fully completed at least one of the three psychometric instruments administered, giving an overall effective response rate of 69%. This response rate was higher than the typical rate of 61% reported for mailed surveys of practitioners (Cummings et al., 2001), and similar to the mean rate of 68% reported in previous studies using surveys mailed to American practitioners (Cull et al., 2005).

The mean age of participants was 44 years (range, 22–64 years, *SD* = 11). In the entire sample, 164 participants (26%) identified themselves as physicians (73 men and 90 women), 286 (46%) as nurses (23 men and 259 women), and the remaining 178 participants (28%) declined to answer this question. The score distribution, descriptive statistics, and reliability of the three instruments used in this study are described in **Table 1**.

**Table 1:** Descriptive statistics and psychometric reliability of scales of collateral effects, loneliness, and empathy in a sample of Spanish healthcare professionals.

Instruments	*n*	PR	AR	Mdn	*M*	*SD*	Reliability
**Collateral effects**	435	13–91	13–91	38	40	16	0.93
Somatization	461	5–35	5–35	13	14	7	0.85
Exhaustion	461	4–28	4–28	14	14	6	0.90
Alienation	448	4–28	4–28	10	11	6	0.87
**Loneliness**	433	15–105	15–77	28	31	14	0.88
Family loneliness	451	5–35	5–26	6	9	4	0.77
Romantic loneliness	453	5–35	5–35	9	12	8	0.88
Social loneliness	463	5–35	5–29	9	11	5	0.81
**Empathy**	484	20–140	67–140	119	118	12	0.83

*n, sample size; PR, possible range; AR, actual range; Mdn, Median; *M*, mean; *SD*, standard deviation.*

An association between positive human connections and occupational well-being was confirmed by measures of empathy and loneliness, assessed by the first two hypotheses. Pearson’s correlation analysis confirmed an inverse association between empathy and the three collateral effects measured by the first hypothesis: somatization (*r* = -0.16; *p* < 0.001), exhaustion (*r* = -0.14; *p =* 0.003), and work alienation (*r* = -0.16; *p* < 0.001). In contrast, loneliness exhibited a positive association with the three collateral effects (*r* = 0.22; *p <* 0.001) confirming the second hypothesis. This also occurred for each of the three dimensions of loneliness that were analyzed as is shown in **Table 2**.

**Table 2:** Pearson’s correlation analysis among collateral effects, loneliness, empathy, and age in Spanish healthcare professionals.

Indicators	Collateral effects			
	Global	Somatization	Exhaustion	Alienation
**Professional competence**				
Empathy	-0.16***	-0.16***	-0.14**	-0.16***
**Psychosocial indicators**				
Loneliness				
Global	0.22***	0.18***	0.18***	0.21***
Family loneliness	0.24***	0.20***	0.18***	0.23***
Romantic loneliness	0.12**	0.10*	0.09*	0.12**
Social loneliness	0.19***	0.13**	0.17***	0.17***
Age	-0.03	-0.13**	-0.01	0.07
Family burden	0.03	0.00	0.03	0.06
**Work indicators**				
Professional experience (years)	-0.08	-0.18***	-0.08	0.02
Work dedication to (hours):				
Clinics	0.00	-0.01	0.02	0.02
Management	0.08	0.07	0.07	0.04
Research	-0.09	-0.12**	-0.08	-0.06
Monthly salary	-0.15***	-0.20***	-0.13**	-0.05

*^∗^p < 0.05; ^∗∗^*p* < 0.01; ^∗∗∗^*p* < 0.001.*

**FIGURE 1:**
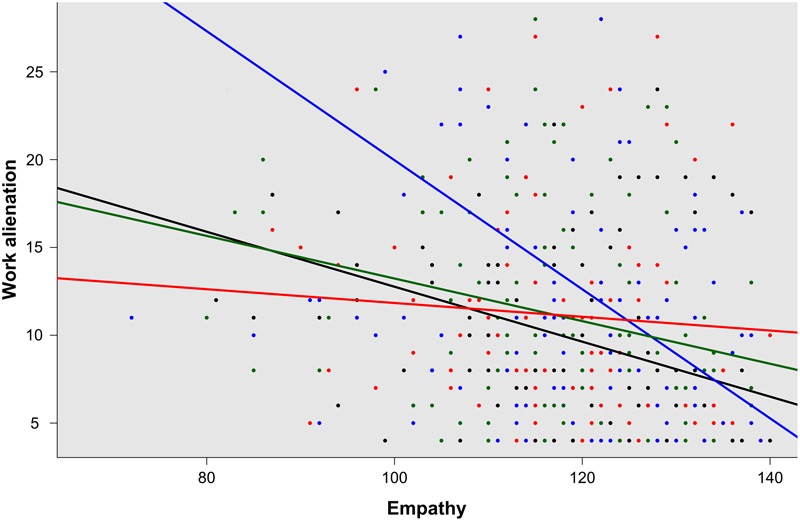
Regression analysis for “occupation by sex” groups, male physicians (black), women physicians (green), male nurses (blue), and women nurses (red), according to alienation by empathy (*p* = 0.03).

Regarding the third hypothesis, the entire sample was divided into four groups according to the combination of occupation and sex variables. Comparative analyses demonstrated differences among “occupation by sex” groups for collateral effects (*p* = 0.03), and for empathy (*p* = 0.04), but not for loneliness (*p* = 0.84), confirming the third hypothesis tested. Moreover, *post hoc* analysis confirmed that female nurses suffered more collateral effects than male physicians (*p* = 0.038). A similar analysis also showed that male nurses showed a tendency, even if not meaningful, toward a higher empathetic orientation than male physicians (*p* = 0.053). These findings are summarized in **Table 3**. Regression analysis with interaction terms for each of the collateral effects measured demonstrated that there were statistically significant differences in association with alienation according to “occupation by sex” (*p* = 0.03) (**Figure 1**).

**Table 3:** Summary result of Kruskal–Wallis test to compare “occupation by sex” groups in a sample of Spanish healthcare professionals (*N* = 450).

Occupation by sex	*n*	Collateral effects			Loneliness			Empathy		
		*M*	*SD*	*p*	*M*	*SD*	*p*	*M*	*SD*	*p*
**Physicians**				0.03			0.84			0.04
Men	73	35	16		33	14		115	13	
Women	91	39	16		31	14		120	11	
**Nurses**										
Men	23	39	17		32	15		123	9	
Women	259	41	16		32	14		118	13	

*n, sample size; *M*, mean; *SD*, standard deviation; *p, p*-value.*

**FIGURE 2:**
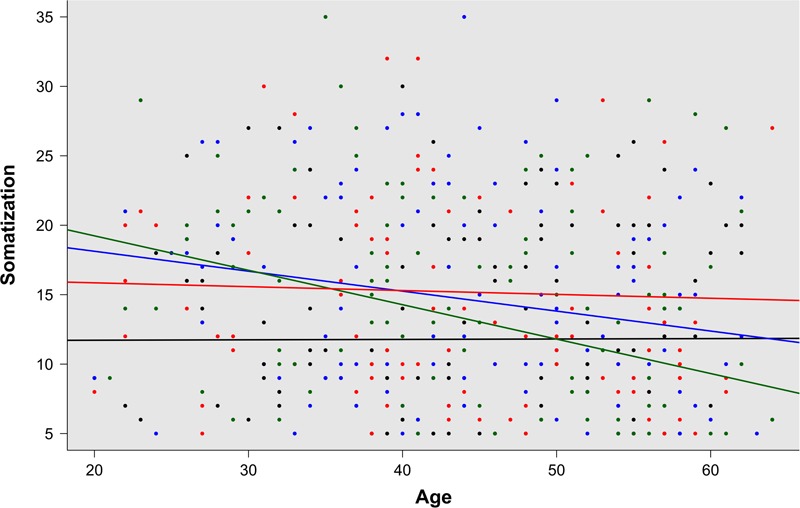
Regression analysis for “occupation by sex” groups, male physicians (black), women physicians (green), male nurses (blue), and women nurses (red), according to somatization by age (*p* = 0.04).

Regarding the other factors assessed (age, family burden, professional experience, work dedication, and salary), an inverse association was identified between age and somatization (*r* = -0.13; *p* = 0.006). After controlling for the effect of age, a similar association was found between somatization and professional experience (*r* = -0.18; *p* < 0.001) by partial correlation analysis. Statistically significant differences were also observed between somatization and age according to “occupation and sex” in regression analysis of the interaction terms (**Figure 2**). Finally, work dedication to research activities (*r* = -0.12; *p* = 0.012) and salary (*r* = -0.20; *p* < 0.001) appeared to be inversely related to somatization. Salary was inversely associated with exhaustion (*r* = -0.13; *p* = 0.007). Neither family burden, nor work dedication to clinics or management activities were associated with any of the three collateral effects measured. With the exception of empathy and age (see above), no differences were observed in the associations according to “occupation by sex” group. A summary of these findings is presented in **Table 2**.

## Discussion

The range observed for the Cronbach’s alpha coefficients obtained in this study was between 0.77 and 0.93. These values are higher than the recommendation of the American Educational Research Association of 0.70, confirming the adequate psychometric reliability of all instruments used in a Spanish clinical context. These findings are also slightly superior to those previously reported for empathy (Hojat et al., 2002; Alcorta-Garza et al., 2016), loneliness (DiTommaso et al., 2004; Yárnoz Yaben, 2008), and collateral effects (Blanch et al., 2010).

### Human Connections and Occupational Well-being of Healthcare Professionals

Regarding characterization of the positive influence of human connections in the promotion of the occupational well-being of healthcare professionals by measurement of empathy, the findings observed in this study are in consonance with those related to the reported positive effects of “relational coordination” among healthcare providers, patients, and their families (Gittell et al., 2000, 2013); and more recently, with those reported in Spanish (Yuguero-Torres et al., 2015, 2017) and Latin American healthcare professionals (Marilaf-Caro et al., 2017; San-Martín et al., 2017), regarding the positive role that empathy plays in the promotion of occupational well-being. Moreover, the findings of this study confirm the important role of empathy in the prevention of somatization (*r* = -0.16; *p* < 0.001), exhaustion (*r* = -0.14; *p* = 0.003), and work alienation (*r* = -0.16; *p* < 0.001), in Spanish healthcare institutions. Furthermore, the findings of this study related to loneliness, as an indicator of social skills deficit, are consistent with the associations previously described in physicians-in-training and nurses between deficit of social skills and burnout and mental health problems, such as emotional exhaustion, emotional detachment, and dehumanization (Pereira-Lima and Loureiro, 2015; Marilaf-Caro et al., 2017). This study provides new evidence clarifying the important role that an absence of positive human connections (measured as loneliness) has in the perception of work-related stress. This role is even more significant than others that have traditionally been focused on, such as family burden or time dedicated to work activities. Moreover, the findings of this study underline the importance for healthcare workers of taking advantage of their family and occupational environments as healthy sources of distraction and personal fulfillment.

### Characterizing Factors Influencing Empathy, Loneliness, and Work Stress

In this study, statistically significant differences were found in collateral effects and empathy, but not in loneliness, when sex, occupation, and the combination of both variables, were compared. These different outcomes can be explained by the conceptualization of loneliness. According to some authors (DiTommaso and Spinner, 1997; DiTommaso et al., 2004), loneliness can be defined as a multidimensional, domain-specific experience. Consequently, despite different types of loneliness sharing a common core, deficits in different relationships and the associated consequences of becoming lonely in a particular relationship domain, such as family, romantic, or social domains, can be qualitatively different (DiTommaso et al., 2004). Based on this conceptualization, it would be expected to find no differences in the perception of loneliness according to occupation, sex, or the combination of both, as observed in this study. Hence, the experience of loneliness and its negative consequences on health and well-being, can occur in all healthcare professionals.

In contrast, comparative and *post hoc* analyses performed for work stress and empathy measures revealed that female nurses perceived higher levels of work-related stress compared with other healthcare professionals, while male physicians perceived the lowest levels of work stress. In this study, male physicians working in Spanish healthcare institutions also showed the lowest empathetic orientation, while male nurses showed the highest. Both outcomes reveal important differences according to occupation and sex. This finding is consistent with a recent study performed with healthcare professionals working in Latin American institutions (San-Martín et al., 2017), where the influence of professional roles and social stereotypes on occupational well-being were demonstrated. The findings of this study also reinforce the role and influence that cultural factors have in the health and well-being of healthcare professionals in their workplaces. Several studies have confirmed that this issue is a global problem that does not only affect developing countries (Ogilvie et al., 2007; Muliira et al., 2012; Ochoa and Blanch, 2016; San-Martín et al., 2017).

Work alienation, one of the three collateral effects measured in this study, is directly related to the progressive loss of meaning of the daily activities performed at the workplace (Sarros et al., 2002). Workers who suffer alienation at their workplace tend to express bad humor, low work satisfaction, depersonalization, and frustration because of their work (Blanch et al., 2010). The findings of this study relating to empathy and work alienation, confirm the important role of empathy in the prevention of work alienation. This function is particularly important in those professionals who spend more time with patients, such as nurses. This association between empathy and alienation was demonstrated in a recent study of Chilean nurses working in palliative care and homecare services (Marilaf-Caro et al., 2017). Hence, the findings observed in this study are in accordance with those previously reported in a different cultural context.

The outcomes observed in this study also provide novel information regarding the relationship between empathy and work alienation, according to the combination of occupation and sex (**Figure 1**). Empathy in clinical encounters, as previously mentioned, is a predominantly cognitive (rather than emotional) attribute, that involves the ability to understand (rather than feel) the experiences, concerns, and perspectives of patients, and communicate this understanding (Hojat et al., 2002; Alcorta-Garza et al., 2016; Hojat, 2016). Findings from a large number of gender studies demonstrate that women, both in the general population and in clinical contexts, are more empathetic than men (Hojat, 2016). Several plausible explanations have been given for such differences, including social learning, genetic predisposition, evolutionary origins, and other factors such as hormonal signals, newborn sensitivity to social stimuli and propensity to social interaction, and interpersonal style, verbal ability, aggressive behavior, and caring attitudes (Hojat et al., 2001, 2002; Alcorta-Garza et al., 2016; Hojat, 2016). However, due to the role of empathy in emotional regulation, this attribute also works as a “professional compass” for healthcare professionals when they are required to engage in stressful working situations in clinical encounters. This is a remarkable issue, particularly for nurses, who are more exposed than physicians to these types of situations in their daily work, due to the higher amount of time they are required to spend with patients compared with physicians. Under such circumstances, it is reasonable to assume that that being male or female would introduce an important difference in how the daily patient care workload is handled. According to Wood and Eagly (2010), women more often use communication to enhance interpersonal relationships due to their *communal character*, while men, because of their *agentic character*, tend to use communication to achieve tangible outcomes and exert dominance. Similarly, Hojat (2016, p. 179), argues that “in stressful situations women would tend to express their emotions and talk about the problem to acquire their mates’ support (a communal characteristic), but men often prefer not to talk but rather do something about problems (an agentic characteristic)”. In this sense, the outcomes observed in this study not only confirm the role of empathy in the prevention of work-related stress, but also demonstrate the benefits that this attribute offers for male nurses.

Finally, this study also provides new information regarding the relationship between somatization and age, according to the combination of occupation and sex (**Figure 2**). In 2016, *Frontiers in Psychology* published a review remarking on the positive role that age plays in occupational well-being (Zacher and Schmitt, 2016). In accordance with this work, a recent publication provided new evidence in support of a positive association between aging and occupational well-being, even when work characteristics differed (San-Martín et al., 2017). The findings of this study, related to somatization, provide new evidence in support of both studies (Zacher and Schmitt, 2016; San-Martín et al., 2017); however, after controlling for the effect of age, a similar association was found between somatization and professional experience, indicating that both factors (age and professional experience) facilitate the reduction of somatization. This result is also in accordance with the “successful aging” work model. According to this model, successful aging at work involves a process during which workers maintain or improve favorable work outcomes, such as motivation, performance, and well-being with “increasing age” (Kooij et al., 2008; Zacher, 2015) or, as observed in this study, also “increasing experience.” The differences observed on comparison of the “occupation by sex” groups regarding the relationship between somatization and age, are in consonance with the “role theory” model, relating to the well-being of workers. According to this model, in workers who occupy multiple roles within and outside the work context, the perception and perceived importance of these roles and more specifically, tasks, expectations, and available resources within those roles, changes over time and with age (Ashforth, 2001). This conceptualization could explain why somatization in women physicians falls more drastically as they age, in comparison with the other three groups. It also explains the similar tendency observed in male nurses. It is remarkable that, in both cases, there are emerging work groups that are directly associated with a drastic change of the traditional Spanish stereotypical work roles that used to associate women with nursing and men with medicine. However, not all these changes have been happening in the same way and in the same speed. For instance, according to Bernalte-Martí (2015), in Spain only 15% of the entire population of nurses is composed by males and this situation continues along time. This fact also helps explain the important difference observed in the number of male and female nurses who participated in this study, but also differences related to empathy in male nurses in comparison with their female counterparts. In Spain, males applying to nursing studies may present some particular baseline characteristics such as, strong vocational attitudes and a resilient attitude when handling dominant social and work role stereotypes.

Overall, these findings stress the importance that the establishment of human connections have in the health and welfare of healthcare professionals, and the roles of some cultural factors and professional roles in healthcare workplaces.

## Author Contributions

LV was in charge of the study’s overall design. JS-G was in charge of coordination with participating institutions in Lleida. MS-M and LV performed statistical processing of data. LV and RD-B prepared the draft manuscript. All authors contributed to the presented work, participated in the interpretation and processing of results, and reviewed and approved the final manuscript.

## Conflict of Interest Statement

The authors declare that the research was conducted in the absence of any commercial or financial relationships that could be construed as a potential conflict of interest.
